# Causative variant profile of collagen VI-related dystrophy in Japan

**DOI:** 10.1186/s13023-021-01921-2

**Published:** 2021-06-24

**Authors:** Michio Inoue, Yoshihiko Saito, Takahiro Yonekawa, Megumu Ogawa, Aritoshi Iida, Ichizo Nishino, Satoru Noguchi

**Affiliations:** 1 Department of Neuromuscular Research, National Institute of Neuroscience, National Center of Neurology and Psychiatry (NCNP), 4-1-1 Ogawa-Higashi, Kodaira, Tokyo, 187-8502 Japan; 2 Department of Genome Medicine Development, Medical Genome Center, NCNP, Tokyo, Japan; 3 Department of Clinical Genome Analysis, Medical Genome Center, NCNP, Tokyo, Japan

**Keywords:** Collagen VI-related dystrophy, Ullrich congenital muscular dystrophy, Bethlem myopathy, Sarcolemma-specific collagen VI deficiency, cDNA analysis

## Abstract

**Background:**

Collagen VI-related dystrophy spans a clinical continuum from severe Ullrich congenital muscular dystrophy to milder Bethlem myopathy. This disease is caused by causative variants in *COL6A1*, *COL6A2*, or *COL6A3*. Most reported causative variants are de novo; therefore, to identify possible associated causative variants, comprehensive large cohort studies are required for different ethnicities.

**Methods:**

We retrospectively reviewed clinical information, muscle histology, and genetic analyses from 147 Japanese patients representing 130 families, whose samples were sent for diagnosis to the National Center of Neurology and Psychiatry between July 1979 and January 2020. Genetic analyses were conducted by gene-based resequencing, targeted panel resequencing, and whole exome sequencing, in combination with cDNA analysis.

**Results:**

Of a total of 130 families with 1–5 members with collagen VI-related dystrophy, 120 had mono-allelic and 10 had bi-allelic variants in *COL6A1*, *COL6A2*, or *COL6A3*. Among them, 60 variants were in *COL6A1*, 57 in *COL6A2*, and 23 in *COL6A3*, including 37 novel variants. Mono-allelic variants were classified into four groups: missense (69, 58%), splicing (40, 33%), small in-frame deletion (7, 6%), and large genomic deletion (4, 3%). Variants in the triple helical domains accounted for 88% (105/120) of all mono-allelic variants.

**Conclusions:**

We report the causative variant profile of a large set of Japanese cases of collagen VI-related dystrophy. This dataset can be used as a reference to support genetic diagnosis and variant-specific treatment.

## Background

Collagen VI is an important component of the interstitium in skeletal muscles, and consists of three chains, alpha 1, 2, and 3, which are encoded by *COL6A1, COL6A2*, and *COL6A3* genes, respectively [1]. Causative variants in *COL6A1*, *COL6A2,* or *COL6A3* cause a clinical continuum collectively called ‘collagen VI-related dystrophy’. At the more severe end of the continuum is Ullrich congenital muscular dystrophy (UCMD; OMIM 254090), and patients may have de novo variants or show autosomal recessive inheritance [2–4]. Bethlem myopathy (BM; OMIM 158810) is at the milder end, and patients mostly show autosomal dominant inheritance [4] although autosomal recessive inheritance has been reported [5, 6]. UCMD is the second- and the third- most common CMD in Japan [7] and in the UK [8]. In a study of the population in northern England, prevalence of UCMD was 0.13 cases per 100,000, whilst the prevalence of BM was 0.77 cases per 100,000 [9].

Collagen VI-related dystrophy shows characteristic clinical phenotypes, which include proximal muscle weakness, skin and joint changes, scoliosis, and respiratory failure [1, 10, 11]. Muscle pathology encompasses variable histological changes including fiber size variation, an increased number of internal nuclei, and disproportionately prominent endomysial connective tissue considering the relative scarceness of necrotic and regenerating fibers [4, 12]. We have previously reported two patterns of collagen VI distribution in muscles among patients: completely deficient (CD) or deficient on the sarcolemma but with deposits in the interstitium (sarcolemma-specific collagen VI deficiency: SSCD) [7, 13].

The eventual diagnosis of this disease is made by genetic analysis. Before and in the era of next-generation sequencing (NGS), several studies have demonstrated a genetic spectrum in collagen VI-related dystrophy, showing that a distribution of variants is common across several ethnic backgrounds [7, 11, 14–16]: the most common glycine substitution in the triple helical domain (THD), other missense variants, nonsense variants, splicing variants causing exon-skipping, small in-frame deletion/insertions, and small deletion/insertions causing a premature stop codon. Large genomic deletions spanning multiple exons are rare [10, 17–19]. Recently, a highly recurrent intronic variant in *COL6A1* has been identified [20].

The aim of the present study was to elucidate the causative variant profile of collagen VI-related dystrophy in Japan by comprehensive genetic analysis including cDNA analysis, and to correlate the findings with immunostaining for collagen VI on muscle biopsies.

## Results

**Fig. 1:**
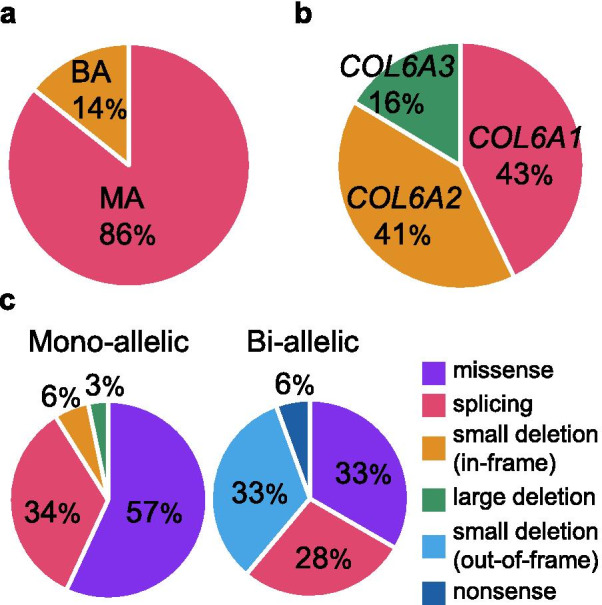
Type and frequency of variants in collagen VI-related dystrophy. The proportion of (**a**) bi-allelic (BA) and mono-allelic (MA) variants, and (**b**) variants in *COL6A1*, *COL6A2*, and *COL6A3*. The frequencies of various types of (**c**) mono-allelic and (d) bi-allelic variants.

**Fig. 2:**
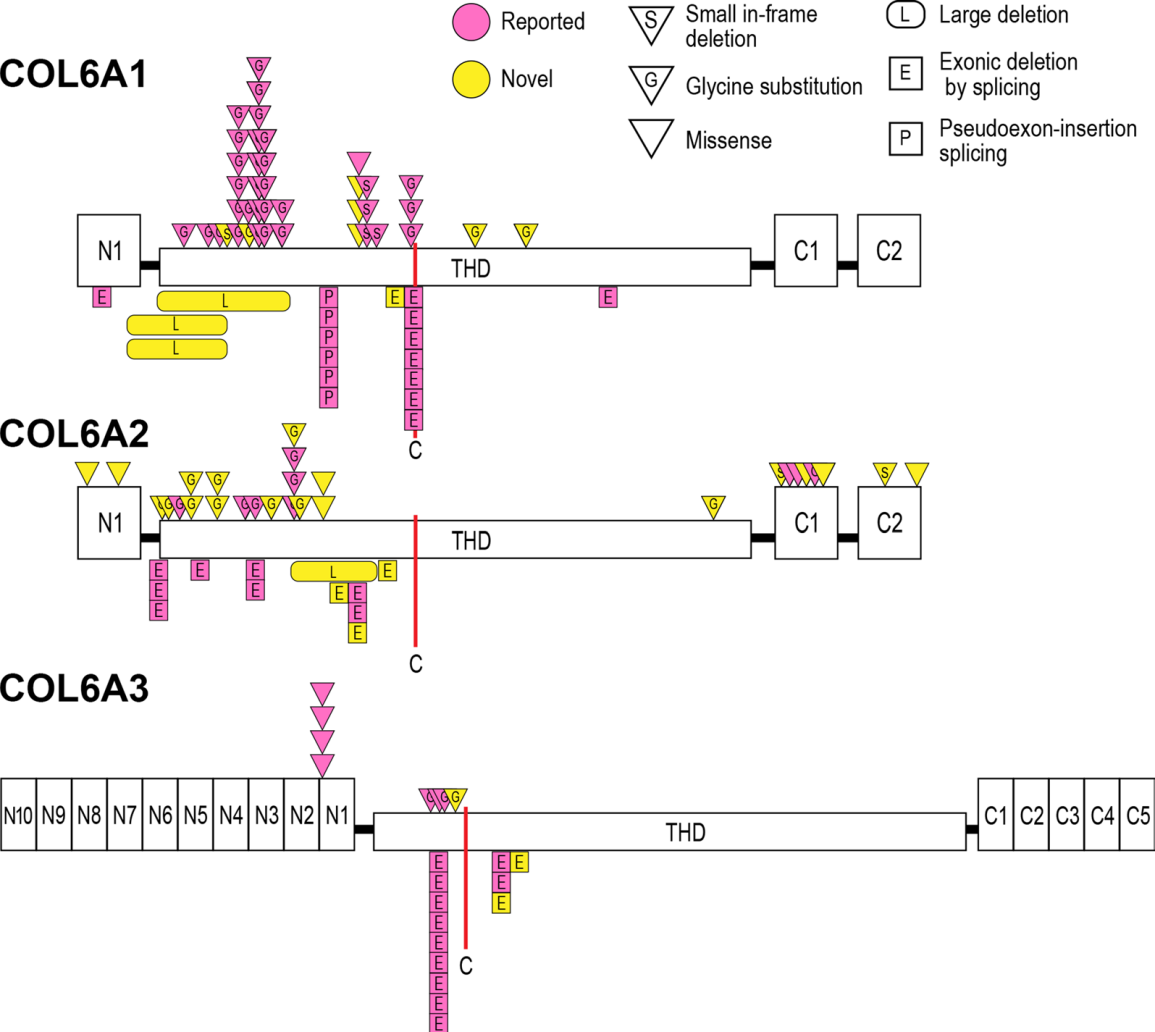
Schematic domain structure of collagen VI polypeptide chains and localization of the identified mono-allelic variants. The identified missense variants and small in-frame deletions are indicated by triangles. Large genomic deletions, exonic deletions by splicing variants, and pseudoexon insertions are indicated by rectangles. Previously reported variants are shown in pink and novel ones in yellow. A single cysteine residue (C) in each triple helical domain (THD) is important for molecular assembly. Most mono-allelic variants are clustered in the N-terminal side of or around the cysteine residue in the THD. (Figure is modified from Lampe et al. [14]).

We identified pathogenic variants in a total of 130 families with collagen VI-related dystrophy, which represented 1–5 members per family, seen at the National Center of Neurology and Psychiatry (NCNP) between July 1979 and January 2020, among them 120 families carried mono-allelic and 10 bi-allelic pathogenic variants (Table 1). One hundred and forty variants were identified, including 37 novel variants in 40 families, and these consisted of 60 allelic variants in *COL6A1*, 57 allelic variants in *COL6A2*, and 23 allelic variants in *COL6A3* (Fig. 1). In 94 families with a mono-allelic variant, this was sporadic without family history (94/130, 72%). Among the 37 novel variants, we identified 24 missense variants, six splicing variants, three small in-frame deletions, three large deletions, and one nonsense variant (Fig. 2).

**Table 1.** Causative variant profile of collagen VI-related dystrophy

Family	Gene	Mono or Bi-allelic	Category	Domain	Nucleotide change	Protein change	Phenotype	COL6 IHC	Inheritance	Report
1	*COL6A1*	BA	Missense	C1	c.1879G>C homozygous	p.G627R	BM	Normal	Recessive	Novel
2-1^a^	*COL6A1*	MA	Splicing	N1	c.428+1G>T	p.Y77_G143del	BM	SSCD	Dominant	[12]
2-2^a^	*COL6A1*	MA	Splicing	N1	c.428+1G>T	p.Y77_G143del	BM	Normal	Dominant	[12]
3	*COL6A1*	MA	Large deletion	THD	c.589-7_804+490del	p.E197_E285del	Intermediate	SSCD	Dominant	Novel
4	*COL6A1*	MA	Large deletion	THD	c.589-7_804+490del	p.E197_E285del	UCMD	SSCD	de novo	Novel
5	*COL6A1*	MA	Large deletion	THD	c.765_903+26del	p.P254_K301del	UCMD	SSCD	de novo	Novel
6	*COL6A1*	MA	Glycine substitution	THD	c.806G>A	p.G269E	UCMD	SSCD	de novo	[28]
7	*COL6A1*	MA	Glycine substitution	THD	c.833G>A	p.G278E	Intermediate	SSCD	de novo	[11]
8	*COL6A1*	MA	Glycine substitution	THD	c.841G>A	p.G281R	Intermediate	Normal	de novo	[14]
9	*COL6A1*	MA	Small deletion	THD	c.845_847del	p.E282del	UCMD	SSCD	de novo	Novel
10	*COL6A1*	MA	Glycine substitution	THD	c.849G>A	p.G284R	UCMD	SSCD	Dominant	[14]
11	*COL6A1*	MA	Glycine substitution	THD	c.850G>A	p.G284R	UCMD	SSCD	de novo	[14]
12	*COL6A1*	MA	Glycine substitution	THD	c.850G>A	p.G284R	UCMD	SSCD	de novo	[14]
13	*COL6A1*	MA	Glycine substitution	THD	c.850G>A	p.G284R	UCMD	SSCD	de novo	[14]
14	*COL6A1*	MA	Glycine substitution	THD	c.850G>A	p.G284R	UCMD	SSCD	de novo	[14]
15	*COL6A1*	MA	Glycine substitution	THD	c.850G>A	p.G284R	UCMD	SSCD	de novo	[14]
16	*COL6A1*	MA	Glycine substitution	THD	c.859G>C	p.G287R	UCMD	NA	de novo	[35]
17	*COL6A1*	MA	Glycine substitution	THD	c.860G>A	p.G287E	UCMD	SSCD	de novo	Novel
18	*COL6A1*	MA	Glycine substitution	THD	c.868G>A	p.G290R	UCMD	SSCD	de novo	[38]
19	*COL6A1*	MA	Glycine substitution	THD	c.868G>A	p.G290R	UCMD	Normal	Dominant	[38]
20	*COL6A1*	MA	Glycine substitution	THD	c.868G>A	p.G290R	Intermediate	SSCD	de novo	[38]
21	*COL6A1*	MA	Glycine substitution	THD	c.868G>A	p.G290R	UCMD	SSCD	de novo	[38]
22	*COL6A1*	MA	Glycine substitution	THD	c.868G>A	p.G290R	Intermediate	NA	de novo	[14]
23	*COL6A1*	MA	Glycine substitution	THD	c.868G>A	p.G290R	Intermediate	SSCD	de novo	[14]
24	*COL6A1*	MA	Glycine substitution	THD	c.868G>A	p.G290R	UCMD	SSCD	de novo	[14]
25	*COL6A1*	MA	Glycine substitution	THD	c.868G>A	p.G290R	Intermediate	SSCD	de novo	[14]
26-1^b^	*COL6A1*	MA	Glycine substitution	THD	c.877G>A	p.G293R	Intermediate	NA	Dominant	[35]
26-2^b^	*COL6A1*	MA	Glycine substitution	THD	c.877G>A	p.G293R	Intermediate	NA	Dominant	[35]
26-3^b^	*COL6A1*	MA	Glycine substitution	THD	c.877G>A	p.G293R	Intermediate	NA	Dominant	[35]
27	*COL6A1*	MA	Glycine substitution	THD	c.877G>A	p.G293R	UCMD	SSCD	de novo	[35]
28	*COL6A1*	MA	Glycine substitution	THD	c.877G>A	p.G293R	UCMD	NA	de novo	[35]
29	*COL6A1*	MA	Glycine substitution	THD	c.877G>A	p.G293R	Intermediate	SSCD	de novo	[35]
30-1^c^	*COL6A1*	MA	Glycine substitution	THD	c.877G>A	p.G293R	BM	NA	Dominant	[35]
30-2^c^	*COL6A1*	MA	Glycine substitution	THD	c.877G>A	p.G293R	BM	NA	Dominant	[35]
31	*COL6A1*	MA	Glycine substitution	THD	c.895G>A	p.G299R	Intermediate	SSCD	de novo	[35]
32	*COL6A1*	MA	Glycine substitution	THD	c.896G>A	p.G299E	Intermediate	SSCD	de novo	[23]
33	*COL6A1*	MA	Splicing	THD	c.930+189C>T	p.K310_G311insTRSTAPRRPLHLEGQGQPPRHPAK	UCMD	SSCD	de novo	[20]
34	*COL6A1*	MA	Splicing	THD	c.930+189C>T	p.K310_G311insTRSTAPRRPLHLEGQGQPPRHPAK	Intermediate	SSCD	de novo	[20]
35	*COL6A1*	MA	Splicing	THD	c.930+189C>T	p.K310_G311insTRSTAPRRPLHLEGQGQPPRHPAK	UCMD	SSCD	de novo	[20]
36	*COL6A1*	MA	Splicing	THD	c.930+189C>T	p.K310_G311insTRSTAPRRPLHLEGQGQPPRHPAK	UCMD	SSCD	de novo	[20]
37	*COL6A1*	MA	Splicing	THD	c.930+189C>T	p.K310_G311insTRSTAPRRPLHLEGQGQPPRHPAK	Intermediate	SSCD	de novo	[20]
38	*COL6A1*	MA	Splicing	THD	c.930+189C>T	p.K310_G311insTRSTAPRRPLHLEGQGQPPRHPAK	UCMD	SSCD	de novo	[20]
39	*COL6A1*	MA	Missense	THD	c.956A>G	p.K319R	Intermediate	SSCD	de novo	Novel
40-1^d^	*COL6A1*	MA	Missense	THD	c.956A>G	p.K319R	BM	SSCD	Dominant	Novel
40-2^d^	*COL6A1*	MA	Missense	THD	c.956A>G	p.K319R	BM	NA	Dominant	Novel
41-1^e^	*COL6A1*	MA	Missense	THD	c.956A>G	p.K319R	BM	SSCD	Dominant	Novel
41-2^e^	*COL6A1*	MA	Missense	THD	c.956A>G	p.K319R	BM	NA	Dominant	Novel
41-3^e^	*COL6A1*	MA	Missense	THD	c.956A>G	p.K319R	BM	NA	Dominant	Novel
41-4^e^	*COL6A1*	MA	Missense	THD	c.956A>G	p.K319R	BM	NA	Dominant	Novel
41-5^e^	*COL6A1*	MA	Missense	THD	c.956A>G	p.K319R	BM	NA	Dominant	Novel
42	*COL6A1*	MA	Missense	THD	c.957G>T	p.K319N	UCMD	SSCD	de novo	[7]
43	*COL6A1*	MA	Splicing	THD	c.958-2A>T	p.G320_K334del	UCMD	SSCD	de novo	Novel
44	*COL6A1*	MA	Small deletion	THD	c.958_966del	p.G320_E322del	UCMD	SSCD	de novo	[7]
45	*COL6A1*	MA	Small deletion	THD	c.958_966del	p.G320_E322del	UCMD	SSCD	de novo	[7]
46	*COL6A1*	MA	Small deletion	THD	c.958_966del	p.G320_E322del	UCMD	SSCD	de novo	[7]
47	*COL6A1*	MA	Small deletion	THD	c.967_975del	p.K324_G326del	UCMD	SSCD	de novo	[7]
48	*COL6A1*	MA	Splicing	THD	c.1003-1G>A	p.G335_D352del	BM	SSCD	de novo	[39]
49	*COL6A1*	MA	Glycine substitution	THD	c.1022G>A	p.G341D	Intermediate	SSCD	de novo	[2]
50-1^f^	*COL6A1*	MA	Glycine substitution	THD	c.1022G>A	p.G341D	BM	SSCD	Dominant	[2]
50-2^f^	*COL6A1*	MA	Glycine substitution	THD	c.1022G>A	p.G341D	BM	NA	Dominant	[2]
51	*COL6A1*	MA	Glycine substitution	THD	c.1022G>T	p.G341V	BM	Normal	Dominant	[18]
52	*COL6A1*	MA	Splicing	THD	c.1056+1G>A	p.G335_D352del	Intermediate	SSCD	de novo	[30]
53	*COL6A1*	MA	Splicing	THD	c.1056+1G>A	p.G335_D352del	Intermediate	SSCD	de novo	[30]
54	*COL6A1*	MA	Splicing	THD	c.1056+1G>A	p.G335_D352del	Intermediate	SSCD	de novo	[30]
55-1^g^	*COL6A1*	MA	Splicing	THD	c.1056+1G>A	p.G335_D352del	BM	Normal	Dominant	[30]
55-2^g^	*COL6A1*	MA	Splicing	THD	c.1056+1G>A	p.G335_D352del	BM	NA	Dominant	[30]
56-1^h^	*COL6A1*	MA	Splicing	THD	c.1056+1G>A	p.G335_D352del	BM	SSCD	Dominant	[30]
56-2^h^	*COL6A1*	MA	Splicing	THD	c.1056+1G>A	p.G335_D352del	BM	NA	Dominant	[30]
57	*COL6A1*	MA	Splicing	THD	c.1056+3A>C	p.G335_D352del	BM	SSCD	de novo	[32]
58	*COL6A1*	MA	Glycine substitution	THD	c.1138G>A	p.G380R	UCMD	SSCD	de novo	Novel
59	*COL6A1*	MA	Glycine substitution	THD	c.1255G>A	p.G419S	BM	Normal	Dominant	Novel
60	*COL6A1*	MA	Splicing	THD	c.1461+4A>G	p.G467_E487del	UCMD	Normal	de novo	[28]
61	*COL6A2*	BA	Splicing Splicing	THD THD-C1	c.1270-1G>C c.1771-3C>G	p.G424_K444del p.G591fs	UCMD	CD	Recessive	[7]
62	*COL6A2*	BA	Splicing	THD	c.1572+1G>C homozygous	p.G508_P524del	UCMD	CD	Recessive	[7]
63	*COL6A2*	BA	Splicing Small deletion	THD C1	c.1770+5G>A c.2267_2272del	p.G562_T573del p.A756_I757del	UCMD	SSCD	Recessive	[13] [13]
64	*COL6A2*	BA	Splicing Small deletion	THD-C1 C1	c.1771-2A>T c.2279_2280del	p.G591fs p.D761fs	BM	CD	Recessive	[7]
65-1^i^	*COL6A2*	BA	Missense Missense	C1 C2	c.2093C>T c.2927T>C	p.A698V p.L976S	BM	SSCD	Recessive	Novel Novel
65-2^i^	*COL6A2*	BA	Missense Missense	C1 C2	c.2093C>T c.2927T>C	p.A698V p.L976S	BM	NA	Recessive	Novel Novel
66	*COL6A2*	BA	PTC Missense	C2	c.2386A>T c.2584C>T	p.K796X p.R862W	UCMD	SSCD	Recessive	Novel, [40]
67	*COL6A2*	BA	Small deletion	C1-C2 THD	c.2678_2700del homozygous	p.P893fs	UCMD	CD	Recessive	[7]
68	*COL6A2*	BA	Missense Small deletion	N1	c.2488C>T c.1487_1512del	p.R830W p.R498fs	BM	SSCD	Recessive	[5, 41]
69	*COL6A2*	MA	Missense	N1	c.167G>A	p.S56N	BM	SSCD	de novo	Novel
70	*COL6A2*	MA	Missense	THD	c.565G>A	p.A189T	BM	Normal	de novo	Novel
71	*COL6A2*	MA	Splicing	THD	c.736-1G>A	p.C246_K267del	UCMD	SSCD	de novo	[42]
72	*COL6A2*	MA	Glycine substitution	THD	c.785G>T	p.G262V	BM	SSCD	Dominant	Novel
73	*COL6A2*	MA	Splicing	THD	c.801+1G>T	p.C246_K267del	UCMD	SSCD	de novo	[16]
74	*COL6A2*	MA	Splicing	THD	c.801+2T>C	p.C246_K267del	UCMD	SSCD	de novo	[3]
75	*COL6A2*	MA	Glycine substitution	THD	c.802G>T	p.G268C	UCMD	SSCD	de novo	Novel
76	*COL6A2*	MA	Glycine substitution	THD	c.812G>A	p.G271D	Intermediate	SSCD	de novo	[7]
77	*COL6A2*	MA	Glycine substitution	THD	c.820G>A	p.G274S	Intermediate	SSCD	Dominant	Novel
78	*COL6A2*	MA	Glycine substitution	THD	c.821G>A	p.G274D	Intermediate	SSCD	de novo	Novel
79	*COL6A2*	MA	Glycine substitution	THD	c.838G>C	p.G280R	Intermediate	SSCD	de novo	Novel
80	*COL6A2*	MA	Glycine substitution	THD	c.839G>A	p.G280D	BM	SSCD	de novo	Novel
81	*COL6A2*	MA	Splicing	THD	c.855+1G>A	p.G268_Q285del	UCMD	SSCD	de novo	[35]
82	*COL6A2*	MA	Splicing	THD	c.856-2A>G	p.G286_K309del	UCMD	SSCD	Dominant	[7]
83-1^j^	*COL6A2*	MA	Splicing	THD	c.856-2A>G	p.G286_K309del	BM	SSCD	Dominant	[7]
83-2^j^	*COL6A2*	MA	Splicing	THD	c.856-2A>G	p.G286_K309del	BM	NA	Dominant	[7]
84	*COL6A2*	MA	Glycine substitution	THD	c.866G>A	p.G289D	BM	SSCD	Dominant	[8]
85	*COL6A2*	MA	Glycine substitution	THD	c.875G>T	p.G292V	UCMD	SSCD	de novo	[7]
86	*COL6A2*	MA	Glycine substitution	THD	c.893G>A	p.G298E	UCMD	SSCD	de novo	Novel
87	*COL6A2*	MA	Large deletion	THD	c.900+102_1000-43del	p.G301_K333del	UCMD	SSCD	de novo	Novel
88	*COL6A2*	MA	Glycine substitution	THD	c.901G>T	p.G301C	UCMD	SSCD	de novo	[7]
89	*COL6A2*	MA	Glycine substitution	THD	c.902G>T	p.G301V	Intermediate	SSCD	de novo	Novel
90	*COL6A2*	MA	Glycine substitution	THD	c.902G>A	p.G301D	UCMD	SSCD	de novo	[7]
91	*COL6A2*	MA	Glycine substitution	THD	c.902G>A	p.G301D	UCMD	SSCD	de novo	[7]
92	*COL6A2*	MA	Glycine substitution	THD	c.911G>T	p.G304V	BM	SSCD	de novo	Novel
93	*COL6A2*	MA	Missense	THD	c.943G>A	p.D315N	UCMD	SSCD	de novo	Novel
94	*COL6A2*	MA	Missense	THD	c.943G>A	p.D315N	BM	Normal	Dominant	Novel
95	*COL6A2*	MA	Splicing	THD	c.950_954+8del	p.G310_K318del	UCMD	SSCD	de novo	Novel
96	*COL6A2*	MA	Splicing	THD	c.955-2A>C	p.G319_K333del	UCMD	NA	de novo	Novel
97	*COL6A2*	MA	Splicing	THD	c.955-2A>G	p.G319_K333del	UCMD	SSCD	de novo	[14]
98	*COL6A2*	MA	Splicing	THD	c.955-2A>G	p.G319_K333del	Intermediate	SSCD	de novo	[14]
99-1^k^	*COL6A2*	MA	Splicing	THD	c.1053+1G>A	p.G334_ R351del	BM	SSCD	Dominant	Novel
99-2^k^	*COL6A2*	MA	Splicing	THD	c.1053+1G>A	p.G334_ R351del	BM	NA	Dominant	Novel
100	*COL6A2*	MA	Glycine substitution	THD	c.1664G>A	p.G555E	UCMD	SSCD	de novo	Novel
101	*COL6A2*	MA	Small deletion	C1	c.1858_1860del	p.I620del	UCMD	SSCD	de novo	Novel
102	*COL6A2*	MA	Missense	C1	c.1861G>A	p.D621N	BM	SSCD	Dominant	[2]
103	*COL6A2*	MA	Missense	C1	c.1870G>A	p.E624K	BM	Normal	de novo	[42]
104	*COL6A2*	MA	Missense	C1	c.2192C>G	p.T731R	BM	SSCD	de novo	Novel
105	*COL6A2*	MA	Glycine substitution	C1	c.2197G>A	p.G733R	BM	SSCD	de novo	[43]
106	*COL6A2*	MA	Missense	C1	c.2271C>G	p.I757M	BM	Normal	de novo	Novel
107	*COL6A2*	MA	Small deletion	C2	c.2741_2743del	p.F914del	BM	SSCD	de novo	Novel
108	*COL6A2*	MA	Missense	C2	c.2978G>A	p.R993H	BM	SSCD	de novo	Novel
109	*COL6A3*	BA	Small deletion Small deletion	N1 C3	c.5692delG c.8737delG	p.V1898fs p.A2913fs	UCMD	CD	Recessive	[7]
110	*COL6A3*	MA	Missense	N1	c.5525G>A	p.G1842E	BM	SSCD	Dominant	[12]
111	*COL6A3*	MA	Missense	N1	c.5525G>A	p.G1842E	BM	SSCD	Dominant	[12]
112-1^l^	*COL6A3*	MA	Missense	N1	c.5525G>A	p.G1842E	BM	NA	Dominant	[12]
112-2^l^	*COL6A3*	MA	Missense	N1	c.5525G>A	p.G1842E	BM	NA	Dominant	[12]
113	*COL6A3*	MA	Missense	THD	c.5525G>A	p.G1842E	BM	Normal	Dominant	[12]
114	*COL6A3*	MA	Splicing	THD	c.6157-2A>G	p.G2053_P2070del	UCMD	SSCD	de novo	[7]
115	*COL6A3*	MA	Splicing	THD	c.6157-2A>G	p.G2053_P2070del	Intermediate	SSCD	de novo	[7]
116	*COL6A3*	MA	Glycine substitution	THD	c.6158G>T	p.G2053V	UCMD	SSCD	de novo	[15]
117-1^m^	*COL6A3*	MA	Missense	THD	c.6199G>A	p.E2067K	BM	SSCD	Dominant	[28]
117-2^m^	*COL6A3*	MA	Missense	THD	c.6199G>A	p.E2067K	BM	Normal	Dominant	[28]
118	*COL6A3*	MA	Splicing	THD	c.6210+1G>A	p.G2053_P2070del	UCMD	SSCD	de novo	[25]
119	*COL6A3*	MA	Splicing	THD	c.6210+1G>A	p.G2053_P2070del	UCMD	SSCD	de novo	[25]
120	*COL6A3*	MA	Splicing	THD	c.6210+1G>A	p.G2053_P2070del	UCMD	SSCD	de novo	[25]
121	*COL6A3*	MA	Splicing	THD	c.6210+1G>A	p.G2053_P2070del	UCMD	SSCD	de novo	[25]
122	*COL6A3*	MA	Splicing	THD	c.6210+1G>A	p.G2053_P2070del	Intermediate	SSCD	de novo	[25]
123	*COL6A3*	MA	Splicing	THD	c.6210+1G>A	p.G2053_P2070del	UCMD	SSCD	de novo	[25]
124	*COL6A3*	MA	Splicing	THD	c.6210+2T>A	p.G2053_P2070del	UCMD	SSCD	de novo	[7]
125	*COL6A3*	MA	Glycine substitution	THD	c.6212G>A	p.G2071D	UCMD	SSCD	de novo	[8]
126	*COL6A3*	MA	Glycine substitution	THD	c.6247G>T	p.G2083C	Intermediate	SSCD	de novo	Novel
127	*COL6A3*	MA	Splicing	THD	c.6309G>A	p.G2095_K2103del	UCMD	SSCD	de novo	Novel
128	*COL6A3*	MA	Splicing	THD	c.6309+1G>A	p.G2095_K2103del	UCMD	SSCD	de novo	[35]
129	*COL6A3*	MA	Splicing	THD	c.6310-2A>G	p.G2104_D2118del	UCMD	SSCD	de novo	Novel
130	*COL6A3*	MA	Splicing	THD	c.6283-1G>T^n^ c.6310-2A>T^n^	p.G2095_K2103delinsNSFLYLPVRLIPSL	Intermediate	SSCD	de novo	[37, 44]

IHC, immunohistochemistry; MA, mono-allelic; BA, bi-allelic; PTC, premature stop codon; THD, triple helical domain; NA, not available; UCMD, Ullrich congenital muscular dystrophy; BM, Bethlem myopathy; CD, complete deficiency; SSCD, sarcolemma-specific collagen VI deficiency

^a^brothers; ^b^26-2 and 26-3 are the sons of 26-1; ^d^40-2 is the father of 40-1; ^e^41-2 and 41-3 are the cousins of 41-1, and 41-4 and 41-5 are the sons of 41-2; ^f^sisters; ^g^55-2 is the son of 55-1; ^h^56-2 is the daughter of 55-1; ^i^brothers; ^j^83-2 is the mother of 83-1; ^k^brothers; ^l^brothers; ^m^117-2 is the mother of 117-1; ^n^two variants on one allele. We used the following transcripts: *COL6A1*, NM_001848; *COL6A2*, NM_001849; *COL6A3*, NM_004369)

Among the ten families with bi-allelic variants, in eight the variants were in *COL6A2*, while the other two each had variants in *COL6A1*, or in *COL6A3*. Six of these ten families had variants producing a premature termination codon or causing aberrant splicing, which leads to in-frame exon skipping in both alleles, and all had UCMD phenotypes. One of the ten families, #66, had a nonsense and a missense variant and also exhibited a UCMD phenotype. The affected individuals of the remaining three families had single nucleotide variants causing non-glycine substitutions and all showed BM phenotypes, although family #68 had a 26 bp-deletion causing a premature termination codon in one allele.

In the 120 families carrying a mono-allelic variant, the variants were as follows: missense (69, 58%), splicing (40, 33%), small in-frame deletion (7, 6%), and large deletion (4, 3%; Table 1). Variants in the THD accounted for 88% (105/120) and glycine substitution accounted for 48% (50/120). The variant c.868G>A (p.G290R) in *COL6A1* was found in eight families, while in 64 (53%) of the mono-allelic variant was unique. With respect to the genotype-phenotype correlation, the majority (82%, 86/105) of families having variants in the THD showed UCMD or intermediate phenotypes, while the majority (93%, 14/15) of families harboring variants outside the THD showed milder phenotypes. It is important to note that all seven families showing the skipping of exon 14 in the THD of *COL6A1* had BM or intermediate phenotypes.

**Fig. 3:**
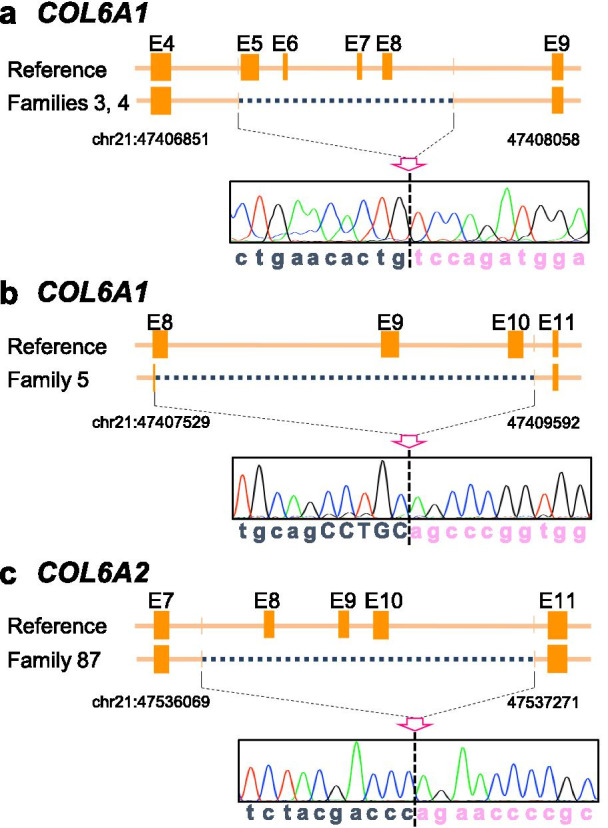
Schematic diagrams and electropherograms at breakpoints of large genomic deletions in *COL6A1* and *COL6A2*. We found a deletion of 216 bp (*COL6A1*) in transcripts in Family #3 and #4, and a deletion of 144 bp (*COL6A1*) and 99 bp (*COL6A2*) in transcripts in Family #5 and #87, respectively. At the genomic level, Family #3 and #4 carried a deletion of 1.2 kb spanning from IVS4-7 to IVS8+490 in *COL6A1* (**a**). The 5′ breakpoint of the 2.1 kb deletion found in Family #5 was located at the sixth base of exon 8 of *COL6A1* and its 3′ breakpoint was at − 43 of intron 10 (**b**). One of the *COL6A2* alleles of Family #87 contained a 1.2 kb deletion extending from IVS7+102 to IVS10-43 (**c**). E: exon sequence. The numbering of genomic positions at the breakpoints are based on the sequence from the Gene Reference Consortium GRCh37/hg19.

Three novel heterozygous multiple exon deletions were detected in four families (Fig. 3). The deletions spanned from exon 5 to exon 8 in *COL6A1* (Family #3 and #4), from exon 8 to exon 10 in *COL6A1* (Family #5), and from exon 8 to exon 10 in *COL6A2* (Family #87). All these large deletions were in-frame and distributed in the THD.

**Fig. 4:**
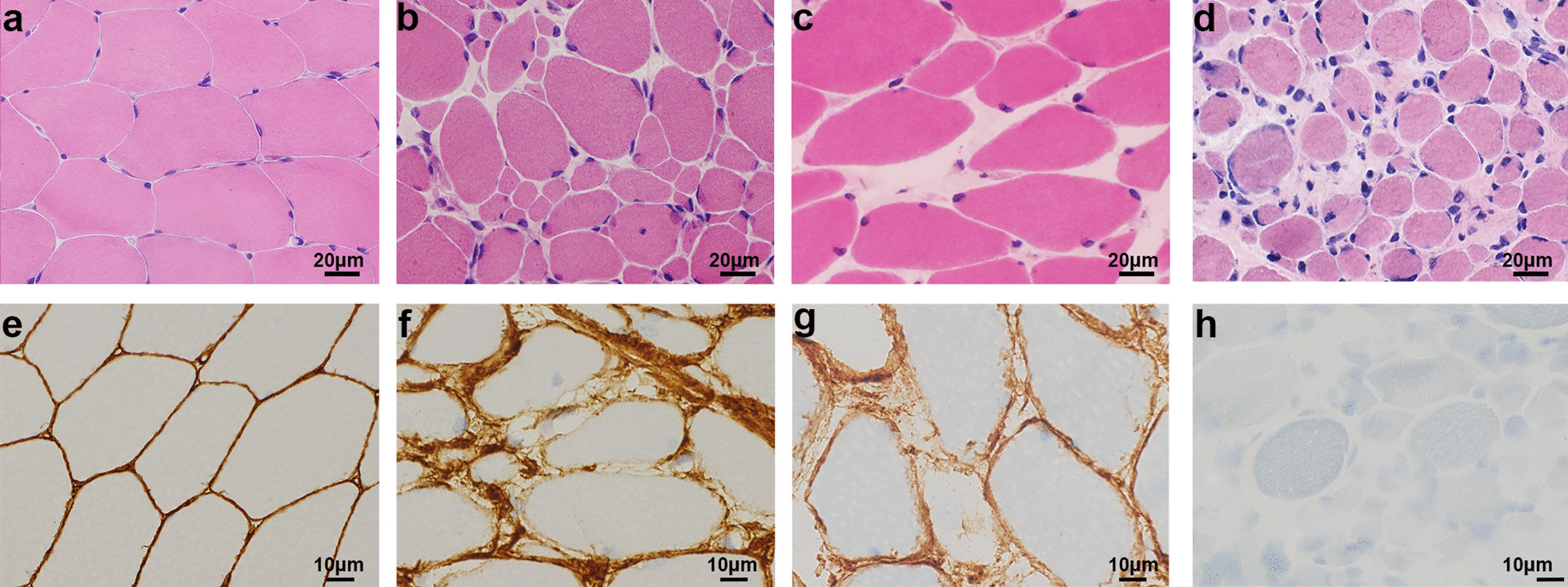
Representative muscle pathology of patients with pathogenic variants in triple helical domains. Histology of muscle from a control individual (**a**, **e**), a member of Family #87 with UCMD and a large genomic deletion (**b**, **f**), a member of Family #72 with BM with a glycine substitution in the triple helical domain (**c**, **g**), and a member of Family #109 with UCMD with bi-allelic small deletions in *COL6A3* (**d**, **h**). Hematoxylin and eosin, scale bar 20 μm. (**a**–**d**) Immunostaining for collagen VI, scale bar 10 μm (**e**–**h**).

**Fig. 5:**
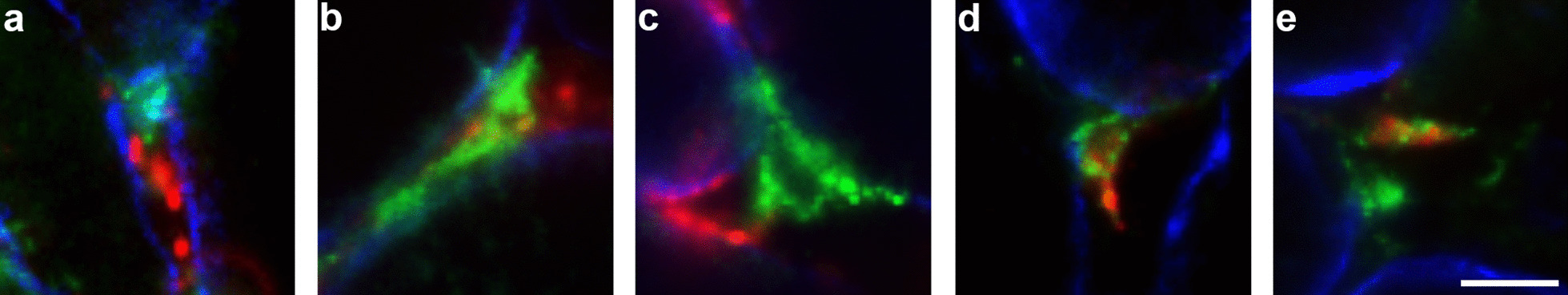
The highly sensitive detection of collagen VI in patients’ muscles showing complete deficiency by routine immunostaining. The highly sensitive immunofluorescence staining for collagen VI (green), PDGFRα (red), and laminin α2 (blue) in muscles of patients showing complete collagen VI deficiency (**a**, Family #64; **b**, Family #67; **c**, Family #109; **d**, Family #61; **e**, Family #62). Scale bar, 10 μm. Highly magnified immunofluorescence images showed that collagen VI formed small deposits in the extracellular space in muscles from patients with truncated variants in both alleles (**a**–**c**), while in patients with an in-frame deletion in at least one allele, the collagen VI was retained within mesenchymal cells (**d**, **e**).

We performed immunostaining for collagen VI in muscle biopsies from 125 affected individuals in 123 families. In 115 patients with a mono-allelic variant, 91% (92/101) with the variant within and 71% (10/14) with the variant outside the THD showed SSCD. Even the biopsies from families harboring multiple exon deletions showed the typical SSCD staining pattern, suggesting dominant-negative effect of those variants (Fig. 4). Among the ten families having bi-allelic variants, five showed a CD pattern, while the five families carrying missense variant(s) showed a SSCD or a normal pattern. Observation at high magnification using immunofluorescence staining revealed trace amounts of extracellular collagen VI in the muscle biopsies of three families with CD (Family #64, #67, and #109), while collagen VI was retained within the mesenchymal cells in two families (#61 and #62; Fig. 5).

We reviewed all available muscle imaging data (34 families including 23 cases and 24 cases tested by MRI and CT, respectively. Thirteen cases were tested by both modalities). At least one of three typical findings in collagen VI-related dystrophy (tigroid or outside in pattern in the vastus lateralis; target sign in the rectus femoris; a hyperintense rim between the soleus and gastrocnemius) [21] was seen in 85% (29/34) of the families. Among 29 families had mono-allelic variants in the THD, 86% (25/29) of these had typical imaging findings. Three in four families (75%) with a mono-allelic variant outside the THD. In families with bi-allelic variants, the imaging data was available in only family, who showed typical imaging findings.

## Discussion

We have elucidated the causative variant profile of collagen VI-related dystrophy in Japan (Table 1). Furthermore, we report 37 novel variants in 40 families, comprising 24 missense, six splicing, three small in-frame deletion, three large genomic deletion, and one nonsense. From the genetic information, we have established the causative variant profile of the largest cohort at a single center as far as we are aware. The majority of the variants were mono-allelic (86%, 120/140), and 67% (94/140) of them were likely to be de novo because the parents of the patients were not apparently affected and their DNAs were not available, as has previously been described [11, 14, 15, 22–24]. Therefore, our causative variant profile may be useful as a reference for diverse ethnicities. Given that all cases with collagen VI-related dystrophy in this cohort were sent to our center from hospitals in Japan, we calculated the occurrence of severe UCMD in Japan as 1.63 cases per year and estimated that about 70% of collagen VI-related dystrophy were diagnosed at our center, which is an estimated incidence of 0.20 in 100,000 births, higher than that found for northern England (0.13/100,000) [9]. This is most likely because of the difference of the diagnostic system between the two countries.

Among the mono-allelic variants, 88% (105/120) were located in the THD. The association between mono-allelic variants in the THD and the SSCD staining pattern (91%, 92/101) may be explained by the fact that tetramers containing dominant mutations in the THD are secreted but cause the impaired ability to form microfibrils and the reduced binding of collagen VI to extracellular matrix [25, 26]. Furthermore, those mono-allelic variants in the THD are associated with UCMD or intermediate phenotype (82%, 86/105). In contrast, mono-allelic variants outside the THD were also associated with SSCD (71%, 10/14) but a BM phenotype (93%, 14/15) (Table 2). However, as shown in the literatures, genotypes cannot be associated with specific phenotypes, with some variants reported to cause both UCMD and BM phenotypes [14–16, 24]. In fact, in our cohort, the families with c.877G>A in *COL6A1*, c.856-2A>G in *COL6A2*, or c.943G>A in *COL6A2* showed a wide range of phenotypes from milder BM to severer UCMD, while conversely the variation in phenotypes of families with c.956A>G or c.1022G>A in *COL6A1* was quite narrow and those families showed BM or intermediate phenotypes.

**Table 2.** Genotype-phenotype correlation of collagen VI-related dystrophy in this study

	Domain	Phenotype	IHC
Mono-allelic	THD	UCMD (55%)	SSCD (91%)
		Intermediate (26%)	
	Outside of the THD	BM (93%)	SSCD (71%)
Bi-allelic	PTC in both alleles	UCMD (100%)	CD (100%)
	Missense/in-frame deletion in at least one allele	UCMD/BM	SSCD (86%)

IHC, immunohistochemistry; PTC, premature stop codon; THD, triple helical domain; UCMD, Ullrich congenital muscular dystrophy; BM, Bethlem myopathy; CD, complete deficiency; SSCD, sarcolemma-specific collagen VI deficiency

In addition, we found four heterozygous large deletions in families with UCMD phenotype. All the deletions were located in the N-terminal side of the cysteine residue important for the assembly of the collagen VI tetramer. This is in accordance with all the reported multiple exon deletions [17, 19, 25, 27–29]. Intriguingly, the deletion in the region containing the cysteine residue caused relatively mild phenotypes in our cohort and in those of previous reports [11, 30–32]. This may be explainable by the mechanism that the loss of the distinctive cysteine residue causes the failure in dimer formation of the mutant COL6A1, resulted in the reduced normal COL6A1 dimer production into 1/4 in amount [31]. On the contrary, deletions of the entire *COL6A2* are reported to show recessively acting loss of function variants [33]. Thus, collagen VI proteins with large genomic deletions in the N-terminal side of the THD, which have the deletions no more than 72 amino acid residues, may act in a dominant-negative fashion and show UCMD or intermediate phenotypes.

In this study, we identified ten families having bi-allelic variants and five and four families showed CD and SSCD collagen VI staining patterns in muscles, respectively. We can presume that families with truncated variants in both alleles will be associated with CD and severe UCMD phenotypes, whilst those with missense variants or in-frame deletions at least in one allele will be associated with SSCD and milder BM phenotypes. In fact, three families with truncated variants in both alleles (CD) and five families with missense or in-frame deletion at least in one allele (SSCD) displayed compatible patterns with the aforementioned presumption, regardless of causative genes. Interestingly, the other two bi-allelic families had in-frame deletion(s) in one and in two alleles, but they showed CD and severe UCMD phenotypes. To explore the mechanism causing the loss of collagen VI in muscles in these families, we observed the trace of collagen VI remaining in their biopsied muscles. In muscles from patients with truncated variants in both alleles, collagen VI formed small deposits in the extracellular space, while in patients with an in-frame deletion in at least one allele, the collagen VI was retained within mesenchymal cells. Thus, we hypothesized that, from those cases with extracellular deposits visible, the truncated collagen VI molecules can form tetramers and be secreted, but the secreted collagen VI will be unstable and degraded extracellularly. On the other hand, in the cases with a retained trace, the in-frame deleted molecules failed to make a tetramer and be secreted. Additional detailed molecular analyses are required to understand the precise mechanism.

The multiple analyses (RNA analysis and immunostaining, reviewing the clinical information) were used for validation of pathogenicity of novel variants. For example, the patients with mono allelic THD variants showed missense or in-frame deletion in transcripts and SSCD staining pattern of collagen VI in muscles, and severe UCMD phenotype. In contrast, the patients with extra-THD variants showed SSCD staining pattern of collagen VI in muscles, and typically milder BM-phenotypes. This information is essentially compatible to the genotype-phenotype correlation in collagen VI-related dystrophy shown in previous reports and adds many examples. The cumulative information further contributes the establishment of the genotype-phenotype database in collagen VI-related dystrophy.

## Conclusion

Our report provides a large causative variant catalog of collagen VI-related dystrophy in Japan, which can be used as a reference for genetic diagnosis and will also be helpful in variant-specific therapy in the future. The majority of causal variants of collagen VI-related dystrophy was mono-allelic de novo, and most of them were located in the THD and associated with SSCD and UCMD or intermediate phenotypes.

## Methods

### Clinical information

This retrospective cohort study was performed on patients seen at the NCNP, a major referral center for muscle disease in Japan, between July 1979 and January 2020. Frozen muscle and blood samples from patients were sent for diagnosis to the NCNP from all over Japan.

Clinically or pathologically suspected collagen VI-related dystrophy with possible pathogenic variants in *COL6A1, COL6A2,* or *COL6A3* was identified in 147 affected individuals in 130 families. Patients with collagen VI-related dystrophy were classified into three categories, UCMD, intermediate and BM, according to phenotypic stratification as previously described [4, 28, 34, 35].

This study was approved by the institutional review boards of the NCNP. All the human materials used in this study were obtained for diagnostic purposes. The patients or their parents provided written informed consent for use of the samples for research.

### Muscle histology

Muscle biopsy samples for histological examination were frozen in isopentane cooled in liquid nitrogen. A set of routine histochemical analyses was performed for diagnosis. When the patients were suspected of having collagen VI-related dystrophy or had elevated serum creatine kinase, immunohistochemistry was performed using standard procedures with an antibody against collagen type VI (VI-26, 1:1000; MP Biomedicals, LLC, Irvine, CA) as previously described [7]. Immunofluorescence staining using standard procedures was performed with antibodies against collagen type VI (VI-26, 1:500; MP Biomedicals), PDGFRα (1:500, Cell Signaling Technology, Danvers MA), and laminin α2 (4H8-2, 1:500; Santa Cruz, Dallas TX)[36].

### Genetic analysis

Genomic DNA was isolated from peripheral blood lymphocytes or muscle specimens using standard techniques. All exons and their flanking intronic regions in *COL6A1*, *COL6A2*, and *COL6A3* were amplified and sequenced directly in 52 families using an ABI PRISM 3130xl Genetic Analyzer (Applied Biosystems, Waltham, MA). Sixty-five families were analyzed using the target resequencing panel for muscular dystrophy because we developed a method for screening gene causative variant in our laboratory since 2014 using Ion PGM NGS [37]. Thirteen families were analyzed by whole exome sequencing because they were initially suspected of having other types of muscular disease.

The splice site-creating variant Chr21:47,409,881 C>T in intron 11 of *COL6A1*, was manually screened by the Sanger method [20].

### cDNA analysis

Total RNA was extracted from frozen muscle using a Total RNA Kit (Nippon Gene, Tokyo, Japan) and cDNA was synthesized with oligo (dT)_20_ primer using SuperScript IV Reverse Transcriptase (Thermo Fisher Scientific, Waltham, MA) using standard techniques [13].

### Identification of pathogenic variants

Novel pathogenic variants were identified using a previously described method [37] with modifications. Briefly, the likely pathogenic variants were defined according to the following criteria: (1) a glycine substitution in the THD; (2) causes exon skipping in the THD; (3) a large genomic deletion; (4) produces a nonsense codon or small insertion/deletion causing a premature stop codon in patients with bi-allelic variants; (5) a missense variant (except a glycine substitution or a substitution outside the THD). If outside the THD, the predicted amino acid substitution was a) predicted to be pathogenic by more than one in silico tool (PolyPhen-2 (http://genetics.bwh.harvard.edu/pph2/), MutationTaster (http://www.mutationtaster.org/), or CADD (http://cadd.gs.washington.edu/)), and/or b) co-segregated with the phenotype within a family. Missense variants were filtered with an allele frequency threshold of < 0.01 in gnomAD (https://gnomad.broadinstitute.org/), NHLBI GO Exome Sequencing Project (http://evs.gs. washington.edu/EVS/), or the integrative Japanese Genome Variation Database (https://ijgvd.megabank.tohoku.ac.jp). The variants identified by target resequencing or whole exome sequencing were confirmed by Sanger sequencing.

## Data Availability

The datasets used and/or analyzed during the current study are available from the corresponding author on reasonable request.

## Abbreviations

BM: Bethlem myopathy
CD: Complete deficiency
SSCD: Sarcolemma-specific collagen VI deficiency
THD: Triple helical domain
UCMD: Ullrich congenital muscular dystrophy
